# Gender difference in relationship between serum ferritin and 25-hydroxyvitamin D in Korean adults

**DOI:** 10.1371/journal.pone.0177722

**Published:** 2017-05-31

**Authors:** Jeong Min Seong, Yo Sang Yoon, Kyu Su Lee, Nan Young Bae, Mi Young Gi, Hyun Yoon

**Affiliations:** 1 Department of Dental Hygiene, College of Health Science, Kangwon National University, Samcheok-si, Gangwon-do, South Korea; 2 Department of Radiological Science, Hanlyo University, Gwangyang-si, jeollanam-do, South Korea; 3 Department of Health Science Graduate School, Chosun University, Gwangju, South Korea; 4 Department of Biomedical Laboratory Science, Kwangyang Health College, Gwangyang-si, Jeollanam-do, South Korea; 5 Department of Nursing, Christian College of Nursing, Gwangju, South Korea; 6 Department of Biomedical Laboratory Science, Hanlyo University, Gwangyang-si, Jeollanam-do, South Korea; University of Alabama at Birmingham, UNITED STATES

## Abstract

**Background:**

The present study was conducted to assess the gender difference in the relationship between serum ferritin and 25-hydroxyvitamin D [25(OH)D] in Korean adults.

**Methods:**

A total of 5,147 adults (2,162 men, 1,563 premenopausal women, and 1,422 postmenopausal women) aged ≥ 20 years from the Korean National Health and Nutrition Examination Survey (KNHANES) data (2012) were analyzed. A covariance test adjusted for covariates was performed for serum ferritin levels in relation to vitamin D status (vitamin D deficiency, 25(OH)D < 10.0 ng/mL; vitamin D insufficiency, 25(OH)D ≥ 10.0, < 20.0 ng/mL; vitamin D sufficiency, 25(OH)D ≥ 20.0 ng/mL).

**Results:**

The key study results were as follows: First, in men, in terms of serum ferritin levels by serum 25(OH)D level after adjusting for age, smoking, alcohol drinking, regular exercise, SBP, DBP, WM. TC, TGs, HDL-C, FPG, Hb, Hct, MCV, and Fe, serum ferritin levels were inversely increased with the increasing of serum 25(OH)D level (*P* = 0.012). Second, in premenopausal women, after adjusting for related variables, serum ferritin levels were increased with the increasing of serum 25(OH)D level (*P* = 0.003). Third, in postmenopausal women, after adjusting for related variables, serum ferritin levels were not significantly increased with the increasing of serum 25(OH)D level (*P* = 0.456).

**Conclusion:**

Serum 25(OH)D level was inversely associated with the serum ferritin levels in men, but was positively associated with the serum ferritin levels in premenopausal women, and was not associated with the serum ferritin levels in postmenopausal women.

## Introduction

Vitamin D is involved in calcium and phosphate absorption in the intestines, whereby it maintains sufficient concentrations of circulating calcium and phosphate levels as well as normal mineralization of bone by providing the minerals to bone-forming sites [1]. Recently, vitamin D has also received attention regarding additional functions concerning its effects on diseases, such as cardiovascular disease, insulin resistance, and iron deficiency anemia [2–5].

Iron is a ubiquitous metal of vital importance to the normal physiologic processes of many organism [6] and it is essential for many metabolic processes, such as oxygen transport, DNA synthesis, and electron transport [7]. Serum ferritin levels are regulated by hepcidin, which plays a role in reducing iron absorption from the intestine [8], and hepcidin is associated with sexual hormones, such as estrogens and testosterone [9,10]. The serum ferritin level reflects iron stores in the body because ferrous iron combined with apoferritin is stored by ferritin in many organisms [11]. A high serum ferritin level is associated with insulin resistance and cardiovascular disease [12,13], and a low serum ferritin level is associated with diseases such as chronic telogen effluvium and iron deficiency anemia [14,15].

Research on the association between vitamin D and ferritin is rare. In addition, the association between vitamin D and ferritin is still being debated because the findings vary across studies. One study reported that vitamin D is positively associated with ferritin [16]. However, other studies have reported that vitamin D is not associated with ferritin [17,18]. In addition, a study reported that the association of vitamin D and ferritin differs in men and women [19]. The Republic of Korea has recently been reported as a country with a severe vitamin D deficiency problem. Among Koreans (age ≥ 10 years), 65.9% of men and 77.7% of women are reported to have a deficiency or insufficiency of vitamin D. In particular, vitamin D deficiency or insufficiency is serious in people aged 20 to 29 years (men, 78.8%; women, 88.3%) [20]. Therefore, our objective in this work was to assess gender differences in the association between vitamin D and ferritin in Korean adults using data from the fifth Korea National Health and Nutrition Examination Survey (KNHANES), which is representative of the population of Korea [21].

## Methods

### Study subjects

KNHANES V-3 data were collected for 1 year (2012), using a rolling sampling survey that involved a complex, stratified, multistage, probability cluster survey of a representative sample of the non-institutionalized civilian population in South Korea. The survey was composed of three parts: a health interview survey, a health examination survey, and a nutrition survey. Each survey was conducted by specially trained interviewers. The interviewers were not provided with any prior information regarding specific participants before conducting the interviews. Participants provided written informed consent to participate in this survey, and we received the data in anonymized form. In the KNHANES V-3 (2012), 8,058 individuals over age 1 were sampled for the survey. Among them, of the 6,221 subjects who participated in the KNHANES V-3, we limited the analyses to adults aged ≥ 20 years. We excluded 874 subjects whose data were missing for important analytic variables such as serum ferritin level, 25(OH)D level, various blood chemistry tests, and information about lifestyle. In addition, we excluded participants who had liver cancer (57 subjects), hepatitis virus B (126 subjects), and hepatitis virus C (17 subjects). Finally, 5,147 subjects (2,162 men, 1,563 premenopausal women, and 1,422 postmenopausal women) were included in the statistical analysis. The KNHANES V-3 study has been conducted according to the principles expressed in the Declaration of Helsinki. (Institutional Review Board No, 2012-01EXP-01-2C). All participants in the survey signed an informed written consent form. Further information can be found in “The KNHANES V-3 (2012) Sample”, which is available on the KNHANES website. The official website of KNHANES (http://knhanes.cdc.go.kr) is currently operating an English-language information homepage. The data of the respective year are available to everyone at the free of charge. If the applicant enters simple subscription process and his/her email address in the official website of KNHANES, the data of the respective year can download to free of charge. If additional information is required, the readers can contact the department responsible for data (Su Yeon Park, sun4070@korea.kr).

### General characteristics and blood chemistry

Research subjects were classified by sex (men, premenopausal, and postmenopausal women), smoking (non-smoker or ex-smoker or current smoker), alcohol drinking (yes or no), and regular exercise (yes or no). In the smoking category, participants who smoked more than one cigarette a day, those who had previously smoked but do not presently smoke, and those who never smoked were classified into the current smoker, ex-smoker, and non-smoker groups, respectively. Alcohol drinking was indicated as “yes” for participants who had consumed at least one glass of alcohol every month over the last year. Regular exercise was indicated as “yes” for participants who had exercised on a regular basis regardless of indoor or outdoor exercise. (Regular exercises was defined as 30 min at a time and 5 times/wk in the case of moderate exercise, such as swimming slowly, doubles tennis, volleyball, badminton, table tennis, and carrying light objects; and for 20 min at a time and 3 times/wk in the case of vigorous exercise, such as running, climbing, cycling fast, swimming fast, football, basketball, jump rope, squash, singles tennis, and carrying heavy objects). Anthropometric measurements included measurement of body mass index (BMI) and waist measurement (WM), as well as final measurements of systolic blood pressure (SBP) and diastolic blood pressure (DBP). Blood chemistries included measurements of total cholesterol (TC), high density lipoprotein cholesterol (HDL-C), triglycerides (TGs), fasting plasma glucose (FPG), 25-hydroxyvitamin D [25(OH)D], serum iron (Fe), total iron binding capacity (TIBC), transferrin saturation (TFS), hemoglobin (Hb), hematocrit (Hct), and mean corpuscular volume (MCV).

### Serum 25(OH)D and ferritin assessments

Blood samples were collected through an antecubital vein after 10–12 h of fasting to assess serum levels of biochemical markers. Serum 25(OH)D levels were measured with a radioimmunoassay (25-hydroxy-vitamin D ^125^ I RIA Kit; DiaSorin, Still Water, MN, USA) using a 1470 Wizard Gamma Counter (Perkin Elmer, Turku, Finland). To minimize the analytical variation, serum 25(OH)D levels were analyzed by the same institute, which carried out a quality assurance program through the analysis period. Serum 25(OH)D levels were classified as either vitamin D deficiency [25(OH)D < 10.0 ng/mL], vitamin D insufficiency [25(OH)D ≥ 10.0, < 20.0 ng/mL], or vitamin D sufficiency [25(OH)D ≥ 20.0 ng/mL] [22]. Concentrations of ferritin were measured using an immunoturbidimetric Assay (IRMA-mat Ferritin; DiaSorin, Still Water, MN, USA) using a 1470 Wizard Gamma Counter (Perkin Elmer, Turku, Finland).

### Statistical analysis

The collected data were statistically analyzed using SPSS WIN version 18.0 (SPSS Inc., Chicago, IL, USA). The distributions of the participant characteristics were converted into percentages, and the successive data were presented as averages with standard deviations. The distribution and average difference in clinical characteristics and iron related indices according to vitamin D were calculated using chi-squared and an analysis of variance (ANOVA). In the case of analysis of covariance test (ANCOVA), the 3 models constructed were: 1) serum 25(OH)D level, age, smoking, alcohol drinking, and regular exercise; 2) serum 25(OH)D level, age, smoking, alcohol drinking, regular exercise, SBP, DBP, WC, and BMI; and 3) serum 25(OH)D level, age, smoking, alcohol drinking, regular exercise, SBP, DBP, WC, BMI, TC, TGs, HDL-C, FPG, Hb, Hct, MCV, and Fe. The significance level for all of the statistical data was set as *P* < 0.05.

## Results

### Clinical characteristics of research subjects

The clinical characteristics of the research subjects are shown in Table 1. Amongst the men (2,162 subjects), the mean of serum 25(OH)D level was 17.94 ± 5.61 ng/mL. According to the classification of vitamin D, 105 (4.9%), 1,374 (63.6%), and 683 (31.5%) subjects were classified as having vitamin D deficiency, vitamin D insufficiency, and vitamin D sufficiency, respectively. The mean of serum ferritin level was 116.23 ± 75.49 μg/L. In premenopausal women (1,563 subjects), the mean of serum 25(OH)D level was 14.96 ± 4.81 ng/mL. According to the classification of vitamin D, 205 (13.1%), 1,153 (73.8%), and 205 (13.1%) subjects were classified as having vitamin D deficiency, vitamin D insufficiency, and vitamin D sufficiency, respectively. The mean of serum ferritin level was 32.47 ± 31.21 μg/L. In postmenopausal women (1,422 subjects), the mean of serum 25(OH)D level was 17.55 ± 5.92 ng/mL. According to the classification of vitamin D, 98 (6.9%), 903 (63.5%), and 421 (29.6%) subjects were classified as vitamin D deficient, vitamin D insufficient, and vitamin D sufficient, respectively. The mean of serum ferritin level was 66.80 ± 44.55 μg/L.

**Table 1:** Clinical characteristics of research subjects.

Variables	Total (n = 5,147)	Men (n = 2,162)	Women (n = 2,985)		P value
			Premenopausal (n = 1,563)	Postmenopausal (n = 1,422)	
Age (years)	51.01 ± 16.34	51.24 ± 16.06	38.77 ± 11.79	64.11 ± 9.20	< 0.001
Current smoker (n/%)	959/18.6%	804/37.2%	105/6.7%	50/3.5%	< 0.001
Alcohol drinker (n/%)	2,500/48.6%	1,446/66.9%	692/44.3%	362/25.5%	< 0.001
Regular exerciser (n/%)	307/6.0%	158/7.3%	69/4.4%	80/5.6%	< 0.001
BMI (kg/m^2^)	23.74 ± 3.40	24.07 ± 3.14	22.71 ± 3.61	24.39 ± 3.30	< 0.001
WM (cm)	81.09 ± 9.77	84.39 ± 8.72	75.64 ± 9.49	82.07 ± 8.98	< 0.001
SBP (mmHg)	119.66 ± 17.09	121.93 ± 15.71	109.86 ± 13.73	126.96 ± 17.55	< 0.001
DBP (mmHg)	75.89 ± 10.46	78.55 ± 10.81	72.19 ± 9.53	75.94 ± 9.63	< 0.001
TC (mg/dL)	189.88 ± 36.17	187.61 ± 35.83	183.45 ± 33.50	200.41 ± 37.24	< 0.001
TGs (mg/dL)	130.33 ± 98.17	148.82 ± 110.16	100.80 ± 89.80	134.66 ± 77.99	< 0.001
HDL-C (mg/dL)	51.74 ± 12.61	48.34 ± 11.55	56.52 ± 12.91	51.67 ± 12.11	< 0.001
FPG (mg/dL)	98.68 ± 22.01	101.16 ± 23.10	92.52 ± 18.17	101.70 ± 22.78	< 0.001
Metabolic syndrome (n/%)	1,316/25.6%	518/24.0%	209/13.4%	589/41.4%	< 0.001
Ferritin (μg/L)	77.14 ± 67.19	116.23 ± 75.49	32.47 ± 31.21	66.80 ± 44.55	< 0.001
Fe (μg/dL)	113.43 ± 47.61	129.96 ± 50.35	100.39 ± 49.53	102.63 ± 33.92	< 0.001
TIBC (μg/dL)	317.66 ± 45.40	310.40 ± 40.27	330.96 ± 52.29	314.07 ± 41.30	< 0.001
TFS (%)	36.47 ± 15.72	42.28 ± 16.21	31.42 ± 15.69	33.19 ± 11.62	< 0.001
Hb (g/dL)	13.99 ± 1.61	15.25 ± 1.25	12.91 ± 1.21	13.25 ± 1.05	< 0.001
Hct (%)	41.68 ± 4.15	44.88 ± 3.33	38.97 ± 3.03	39.81 ± 2.88	< 0.001
MCV (fL)	92.18 ± 4.80	92.95 ± 4.29	90.59 ± 5.59	92.77 ± 4.13	< 0.001
25(OH)D (ng/mL)	16.93 ± 5.63	17.94 ± 5.61	14.96 ± 4.81	17.55 ± 5.92	< 0.001
< 10.0 (n/%)	408/7.9%	105/4.9%	205/13.1%	98/6.9%	< 0.001
≥ 10.0, < 20.0 (n/%)	3,460/66.6%	1,374/63.6%	1,153/73.8%	903/63.5%	
≥ 20.0 (n/%)	1,309/25.4%	683/31.5%	205/13.1%	421/29.6%	

BMI: body mass index, WM: waist measurement, SBP: systolic blood pressure, DBP: diastolic blood pressure, TC: total Cholesterol, TGs: triglycerides, HDL-C: high density lipoprotein cholesterol, FPG: fasting plasma glucose, Fe: serum iron, TIBC: total iron binding capacity, TFS: transferrin saturation, Hb: hemoglobin, Hct: hematocrit, MCV: mean corpuscular volume, 25(OH)D: 25-hydroxyvitamin D

### Clinical characteristics of subjects according to serum 25(OH)D in men, premenopausal, and postmenopausal women

The clinical characteristics of subjects according to serum 25(OH)D level are shown in Tables 2, 3 and 4. In men, variables showing a significant difference in the distribution and the mean value in serum 25(OH)D level were age (*P* < 0.001), current smoker (*P* = 0.001), WM (*P* = 0.009), BMI (*P* < 0.001), TGs (*P* < 0.001), HDL-C (*P* = 0.010), Hb (*P* = 0.002), Hct (*P* = 0.023), MCV (*P* = 0.010), metabolic syndrome (*P* = 0.013), and ferritin (*P* < 0.001). In premenopausal women, variables showing a significant difference in the distribution and the mean value in serum 25(OH)D level were age (*P* < 0.001), regular exerciser (*P* = 0.012), TIBC (*P* = 0.027), TFS (*P* = 0.017), Hb (*P* = 0.001), Hct (*P* = 0.001), and ferritin (*P* < 0.001). However, vitamin D was not associated with metabolic syndrome (*P* = 0.247). In postmenopausal women, variables showing a significant difference in the distribution and the mean value in serum 25(OH)D level were age (*P* < 0.001), BMI (*P* = 0.003), DBP (*P* = 0.036), and FBG (*P* = 0.015). However, vitamin D was not associated with metabolic syndrome (*P* = 0.683) and ferritin (*P* = 0.488).

**Table 2:** Clinical characteristics of subjects according to vitamin D status in men.

Variables	Serum 25(OH)D levels			*P-*value
	Deficiency (< 10.0 ng/mL)	Insufficiency (≥ 10.0, < 20.0 ng/mL)	Sufficiency (≥ 20.0 ng/mL)	
Age (years)	46.72 ± 17.75	49.53 ± 16.15	55.36 ± 14.77	< 0.001
Current smoker (n/%)	50/47.6%	521/37.9%	233/34.1%	0.001
Alcohol drinker (n/%)	67/63.8%	917/66.7%	462/67.6%	0.727
Regular exercise (n/%)	6/5.7%	93/6.8%	59/8.6%	0.251
WM (cm)	82.47 ± 9.99	84.76 ± 8.78	83.94 ± 8.32	0.009
BMI (kg/m^2^)	23.36 ± 3.63	24.26 ± 3.17	23.79 ± 2.95	< 0.001
SBP (mmHg)	123.36 ± 19.28	121.69 ± 15.38	122.22 ± 15.76	0.489
DBP (mmHg)	79.69 ± 12.30	78.83 ± 10.75	77.80 ± 10.65	0.070
TC (mg/dL)	184.32 ± 38.87	188.15 ± 36.16	187.03 ± 34.68	0.504
TGs (mg/dL)	166.75 ± 128.74	155.77 ± 120.63	132.10 ± 78.74	< 0.001
HDL-C (mg/dL)	48.16 ± 12.00	47.80 ± 11.62	49.44 ± 11.29	0.010
FPG (mg/dL)	99.15 ± 22.70	101.51 ± 24.41	100.75 ± 20.30	0.516
Metabolic syndrome (n/%)	31/29.5%	349/25.4%	138/20.2%	0.013
Ferritin (μg/L)	129.09 ± 84.28	119.78 ± 76.49	107.10 ± 71.13	< 0.001
Fe (μg/dL)	131.61 ± 52.12	129.73 ± 47.89	130.15 ± 54.77	0.927
TIBC (μg/dL)	314.76 ± 39.36	310.03 ± 38.92	310.48 ± 42.99	0.510
TFS (%)	42.33 ± 17.21	42.21 ± 15.51	42.42 ± 17.41	0.964
Hb (g/dL)	15.11 ± 1.34	15.32 ± 1.20	15.13 ± 1.25	0.002
Hct (%)	44.54 ± 3.62	45.03 ± 3.21	44.64 ± 3.50	0.023
MCV (fL)	93.04 ± 4.14	92.75 ± 4.16	93.35 ± 4.52	0.010

25(OH)D: 25-hydroxyvitamin D, WM: waist measurement, BMI: body mass index, SBP: systolic blood pressure, DBP: diastolic blood pressure, TC: total Cholesterol, TGs: triglycerides, HDL-C: high density lipoprotein cholesterol, FPG: fasting plasma glucose, Fe: serum iron, TIBC: total iron binding capacity, TFS: transferrin saturation, Hb: hemoglobin, Hct: hematocrit, MCV: mean corpuscular volume.

**Table 3:** Clinical characteristics of subjects according to vitamin D status in premenopausal women.

Variables	Serum 25(OH)D levels			*P-*value
	Deficiency (< 10.0 ng/mL)	Insufficiency (≥ 10.0, < 20.0 ng/mL)	Sufficiency (≥ 20.0 ng/mL)	
Age (years)	38.23 ± 12.08	38.40 ± 11.47	41.31 ± 12.95	0.004
Current smoker (n/%)	17/8.3%	76/6.6%	12/5.9%	0.075
Alcohol drinker (n/%)	81/39.5%	526/45.6%	85/41.5%	0.184
Regular exerciser (n/%)	2/1.0%	61/5.3%	6/2.9%	0.012
WM (cm)	74.35 ± 9.73	75.77 ± 9.55	76.25 ± 8.79	0.088
BMI (kg/m^2^)	22.26 ± 3.69	22.78 ± 3.64	22.78 ± 3.27	0.158
SBP (mmHg)	110.71 ± 13.68	109.75 ± 13.50	109.62 ± 15.06	0.629
DBP (mmHg)	73.19 ± 10.18	72.15 ± 9.53	71.38 ± 8.79	0.151
TC (mg/dL)	180.93 ± 34.67	183.97 ± 33.88	183.04 ± 30.02	0.482
TGs (mg/dL)	107.72 ± 81.32	100.28 ± 93.15	93.77 ± 77.96	0.434
HDL-C (mg/dL)	54.73 ± 13.28	56.79 ± 12.66	56.83 ± 13.80	0.103
FPG (mg/dL)	93.19 ± 15.02	92.43 ± 17.58	93.38 ± 23.57	0.759
Metabolic syndrome (n/%)	35/17.1%	148/12.8%	26/12.7%	0.247
Ferritin (μg/L)	27.34 ± 24.77	31.85 ± 30.32	41.09 ± 39.40	< 0.001
Fe (μg/dL)	94.02 ± 47.90	100.68 ± 47.91	105.13 ± 44.45	0.056
TIBC (μg/dL)	333.51 ± 55.85	332.11 ± 52.35	321.88 ± 47.32	0.027
TFS (%)	29.40 ± 15.55	31.35 ± 15.71	33.79 ± 15.48	0.017
Hb (g/dL)	12.67 ± 1.35	12.92 ± 1.21	13.11 ± 1.05	0.001
Hct (%)	38.32 ± 3.25	38.99 ± 3.00	39.44 ± 2.80	0.001
MCV (fL)	90.30 ± 6.40	90.49 ± 5.55	91.45 ± 4.84	0.056

25(OH)D: 25-hydroxyvitamin D, WM: waist measurement, BMI: body mass index, SBP: systolic blood pressure, DBP: diastolic blood pressure, TC: total Cholesterol, TGs: triglycerides, HDL-C: high density lipoprotein cholesterol, FPG: fasting plasma glucose, Fe: serum iron, TIBC: total iron binding capacity, TFS: transferrin saturation, Hb: hemoglobin, Hct: hematocrit, MCV: mean corpuscular volume.

**Table 4:** Clinical characteristics of subjects according to vitamin D status in postmenopausal women.

Variables	Serum 25(OH)D levels			*P-*value
	Deficiency (< 10.0 ng/mL)	Insufficiency (≥ 10.0, < 20.0 ng/mL)	Sufficiency (≥ 20.0 ng/mL)	
Age (years)	64.72 ± 9.64	63.25 ± 9.14	65.81 ± 9.01	< 0.001
Current smoker (n/%)	18/18.4%	239/26.5%	105/24.9%	0.208
Alcohol drinker (n/%)	3/3.1%	37/4.1%	10/2.4%	0.517
Regular exerciser (n/%)	4/4.1%	58/6.4%	18/4.3%	0.227
WM (cm)	81.89 ± 9.05	82.41 ± 9.17	81.39 ± 8.53	0.152
BMI (kg/m^2^)	24.03 ± 3.10	24.62 ± 3.44	23.98 ± 2.98	0.003
SBP (mmHg)	130.90 ± 20.01	126.89 ± 17.12	126.20 ± 17.78	0.057
DBP (mmHg)	77.54 ± 10.98	76.17 ± 9.60	75.07 ± 9.30	0.036
TC (mg/dL)	198.88 ± 34.48	201.05 ± 37.74	199.42 ± 36.82	0.696
TGs (mg/dL)	145.92 ± 87.55	136.08 ± 82.78	129.02 ± 63.44	0.103
HDL-C (mg/dL)	49.89 ± 10.76	51.70 ± 12.03	52.01 ± 12.58	0.292
FPG (mg/dL)	103.23 ± 23.99	102.79 ± 25.44	99.00 ± 14.94	0.015
Metabolic syndrome (n/%)	41/41.8%	381/42.2%	167/39.7%	0.683
Ferritin (μg/L)	61.68 ± 42.88	67.00 ± 43.97	67.56 ± 46.17	0.488
Fe (μg/dL)	96.57 ± 34.45	103.66 ± 34.67	101.82 ± 32.03	0.123
TIBC (μg/dL)	313.77 ± 37.03	314.17 ± 41.80	313.94 ± 41.25	0.993
TFS (%)	31.15 ± 11.52	33.49 ± 11.86	33.00 ± 11.07	0.153
Hb (g/dL)	13.05 ± 1.05	13.30 ± 0.98	13.21 ± 1.17	0.051
Hct (%)	39.30 ± 2.92	39.92 ± 2.76	39.70 ± 3.12	0.077
MCV (fL)	92.78 ± 7.01	92.73 ± 3.83	92.85 ± 4.74	0.895

25(OH)D: 25-hydroxyvitamin D, WM: waist measurement, BMI: body mass index, SBP: systolic blood pressure, DBP: diastolic blood pressure, TC: total Cholesterol, TGs: triglycerides, HDL-C: high density lipoprotein cholesterol, FPG: fasting plasma glucose, Fe: serum iron, TIBC: total iron binding capacity, TFS: transferrin saturation, Hb: hemoglobin, Hct: hematocrit, MCV: mean corpuscular volume.

### Comparisons of serum ferritin levels according to serum 25(OH)D in men, premenopausal, and postmenopausal women

Comparisons of serum ferritin levels according to serum 25(OH)D level are shown in Table 5. In men, in terms of serum ferritin levels by serum 25(OH)D level for age, smoking, alcohol drinking, regular exercising, SBP, DBP, WM, TC, TGs, HDL-C, FPG, Hb, Hct, MCV, and Fe, serum ferritin levels (M ± SE) were 130.15 ± 7.06 μg/L [95% confidence interval (CI), 116.31–143.99] for vitamin D deficiency, 118.28 ± 1.94 μg/L (95% CI, 114.48–122.09) for vitamin D insufficiency, and 110.63 ± 2.78 μg/L (95% CI, 105.17–116.08) for vitamin D sufficiency, showing that serum ferritin levels were significantly decreased with the increasing of serum 25(OH)D level (*P* = 0.012). However, in premenopausal women, in terms of serum ferritin levels by serum 25(OH)D level after adjusting for related variables, serum ferritin levels (M ± SE) were 28.90 ± 1.96 μg/L (95% CI, 25.07–32.74) for vitamin D deficiency, 32.08 ± 0.82 μg/L (95% CI, 30.48–33.68) for vitamin D insufficiency, and 38.13 ± 1.95 μg/L (95% CI, 34.31–41.94) for vitamin D sufficiency, showing that serum ferritin levels were significantly increased with the increasing of serum 25(OH)D level (*P* = 0.003). In postmenopausal women, in terms of serum ferritin levels by vitamin D after adjusting for related variables, serum ferritin levels (M ± SE) were 62.50 ± 4.29 μg/L (95% CI, 54.09–70.92) for vitamin D deficiency, 66.60 ± 1.41 μg/L (95% CI, 63.82–69.37) for vitamin D insufficiency, and 68.33 ± 2.08 μg/L (95% CI, 64.27–72.43) for vitamin D sufficiency, showing that serum ferritin levels were not significantly increased with the increasing of serum 25(OH)D level (*P* = 0.456).

**Table 5:** Comparisons of serum ferritin levels according to vitamin D status.

	Serum ferritin levels (μg/L)		
	Model 1	Model 2	Model 3
Men (n = 2,162)			
25(OH)D (ng/mL) < 10.0	128.45 ± 7.28 (114.17–142.73)	130.86 ± 7.21 (116.73–144.99)	130.15 ± 7.06 (116.31–143.99)
≥ 10.0, < 20.0	119.64 ± 2.02 (115.69–123.60)	119.14 ± 1.99 (115.23–123.05)	118.28 ± 1.94 (114.48–122.09)
≥ 20.0	107.64 ± 2.88 (101.83–113.12)	108.44 ± 2.85 (102.86–114.02)	110.63 ± 2.78 (105.17–116.08)
*P-*value	0.001	0.001	0.012
Premenopausal (n = 1,563)			
25(OH)D (ng/mL) < 10.0	27.54 ± 2.14 (23.35–31.74)	27.77 ± 2.14 (23.57–31.97)	28.90 ± 1.96 (25.07–32.74)
≥ 10.0, < 20.0	32.05 ± 0.90 (30.28–33.81)	31.97 ± 0.90 (30.21–33.73)	32.08 ± 0.82 (30.48–33.68)
≥ 20.0	39.79 ± 2.14 (35.59–43.98)	39.83 ± 2.14 (35.64–44.02)	38.13 ± 1.95 (34.31–41.94)
*P-*value	< 0.001	< 0.001	0.003
Postmenopausal (n = 1,422)			
25(OH)D (ng/mL) < 10.0	61.99 ± 4.51 (53.15–70.83)	61.76 ± 4.51 (52.92–70.61)	62.50 ± 4.29 (54.09–70.92)
≥ 10.0, < 20.0	67.05 ± 1.49 (64.14–69.97)	66.93 ± 1.49 (64.02–69.85)	66.60 ± 1.41 (63.82–69.37)
≥ 20.0	67.38 ± 2.19 (63.10–71.67)	67.70 ± 2.19 (63.41–71.99)	68.33 ± 2.08 (64.27–72.43)
*P-*value	0.538	0.490	0.456

Model 1 [M ± SE (95% CI)], adjusted for age, smoking, alcohol drinking, and regular exercise; Model 2 [M ± SE (95% CI)], Model 1 further adjusted for SBP, DBP, WM, and BMI; Model 3 [M ± SE (95% CI)], Model 2 further adjusted for, TC, TGs, HDL-C, FPG, Hb, Hct, MCV, and Fe.

## Discussion

The present study investigated the association between serum ferritin levels and vitamin D using data from the fifth KNHANES conducted in 2012. After adjusting for variables, key findings of this study were revealed. Serum 25(OH)D level was inversely associated with serum ferritin levels in men. Conversely, serum 25(OH)D level was positively associated with serum ferritin levels in premenopausal women and was not associated with serum ferritin levels in postmenopausal women (Table 5).

A high prevalence of vitamin D deficiency exists in various populations worldwide and is becoming a serious concern owing to its health issues [1]. Vitamin D is known to prevent cardiovascular disease, insulin resistance, and iron deficiency anemia [4,23,24]. Ferritin, which is an iron storage protein, is found in every cell, such as the liver, spleen, heart, and kidney, and its concentration varies with gender and age [25]. Ferritin is decreased in patients with iron deficiency anemia; in contrast, it is increased in patients with insulin resistance or inflammation [26]. In the present study, ferritin was positively associated with metabolic syndrome and anemia index (Hb and Hct) in men, premenopausal women, and postmenopausal women (S1-S3). Many studies have reported that both serum 25(OH)D levels and ferritin are associated with diseases, such as iron deficiency anemia, insulin resistance, and inflammation [2–4,13,27].

Currently, research on gender differences concerning the association between serum 25(OH)D level and ferritin is rare. A study reported that serum 25(OH)D level correlated positively with ferritin level in women (*r* = 0.160, *P* = 0.009), but not in men (*r* = -0.036, *P* = 0.535) [19]. This result contrasts with the present study that found serum 25(OH)D level was inversely associated with ferritin level in men (*P* = 0.012) but concurs with the finding that serum 25(OH)D level was positively associated with ferritin level in premenopausal women (*P* = 0.003). Although, the present study also found that serum 25(OH)D level was not associated with ferritin level in postmenopausal women (*P* = 0.456).

The gender differences in serum 25(OH)D level and ferritin have some possible explanations. First, sex hormones, such as testosterone and estrogen may be responsible. Serum 25(OH)D level has been positively associated with increased levels of testosterone and estrogen, in men and women [28–30]. Estrogen directly regulates hepatic hepcidin expression through a functional estrogen response element in the promoter region of the hepcidin gene [31]. In premenopausal women, in particular, 17β-estradiol increases iron uptake to compensate for iron loss during menstruation [32]. However, postmenopausal women have an accelerated reduction of estrogens caused by menopause. Therefore, despite the increase in serum 25(OH)D concentration, the ferritin level may be not significantly different in postmenopausal women. However, a study suggested that testosterone increases ferritin by inhibiting hepcidin in men [33]. In contrast, in a large-scale study, Liu and colleagues reported that testosterone is inversely associated with ferritin [34]. Similarly, Chao and colleague reported that testosterone was inversely associated with ferritin in normal weight subjects. However, it was not associated with ferritin in overweight and obese subjects [35].

Second, the role of serum 25(OH)D may vary under certain conditions, such as iron deficiency anemia, insulin resistance, and oxidative stress. As above mentioned, ferritin is down-regulated by hepcidin and decreased in iron deficiency anemia. Vitamin D is associated with alterations of the hepcidin-ferroportin axis in monocytes exposed to lipopolysaccharide and leads to a decrease in pro-hepcidin cytokine, IL-6, and IL-1β [36]. This process has a positive effect in iron deficiency anemia. However, a previous study suggested that ferritin is a useful marker of insulin resistance, and reflects oxidative stress, particularly in men [37]. We were unable to demonstrate that serum 25(OH)D level is negatively associated with ferritin in men. However, we considered that in men, ferritin may be more associated with insulin resistance, inflammation, and oxidative stress than iron deficiency anemia. In the present study, ferritin level was higher in men (116.23 ± 75.49 μg/L) than in premenopausal (32.47 ± 31.21 μg/L) and postmenopausal women (66.80 ± 44.55 μg/L).

Third, the measurement index of vitamin D in the human body may vary. In the vitamin D metabolism pathways, vitamin D3 (D3), formed in the epidermis or obtained from the diet, is solely activated through sequential hydroxylations by CYP27A1 or CYP2R1 at C25, and by CYP27B1 at C1: [D3 → 25(OH)D3 → 1,25(OH)D3] [38]. Most studies have used 25(OH)D3 and 1,25-dihydroxyvitamin D3 [1,25(OH)_2_D] for the measurement of vitamin status and we also used the 25(OH)D3 level in the blood. However, recent studies uncovered novel metabolic pathways in vitamin D metabolism in human serum and tissues. These studies suggested that vitamin D3 acts as a substrate for cytochrome P450scc (CYP11A1), and CYP11A1 hydroxylases D3 to produce 20-hydroxyvitamin D3 and 20,22 dihydroxyvitamin D3 [39–43]. In addition, they reported that these products exert more anti-proliferative, pro-differentiation, and anti-inflammatory effects than 1,25(OH)_2_D or 25(OH)D [42,43]. CYP11A1 plays an important role in the transport pathways for estrogen and testosterone steroidogenesis [44,45] and is also associated with ferritin heavy chains [46]. Therefore, in future studies, gender differences for ferritin and CYP11A1 or novel products such as 20(OH)D3 and 20,22(OH)_2_D3 should be investigated.

Vitamin D plays a role in preventing anemia but also decreases oxidative stress, insulin resistance, and inflammation [23,24,47]. Ferritin is a marker of iron deficiency anemia but may also be a marker of oxidative stress, insulin resistance, and inflammation [37,48]. In iron deficiency anemia, vitamin D may increase ferritin levels. In contrast, in insulin resistance, inflammation, and oxidative stress, vitamin D may decrease ferritin levels. Furthermore, both processes may occur simultaneously. In our study, we could not demonstrate the mechanisms of these processes, but we were able to determine that serum 25(OH)D was positively associated with ferritin in premenopausal women but inversely associated with ferritin in men. In addition, serum 25(OH)D was not associated with ferritin in postmenopausal women. Gender differences exist in lifestyle (physical activity, drinking, and smoking) and disease (cardiovascular disease and immune function) [49,50]. For this reason, some researchers have suggested that medical hypotheses should consider these effects in men and women [51,52].

The present study has some limitations. First, hepcidin is an important determinant of ferritin. However, hepcidin were not employed in the KNHANES V-3 study (2012). Second, serum calcium concentrations and the daily intake volume of vitamin D are important determinants of serum 25(OH)D levels, but the KNHANES V-3 did not measure serum calcium concentrations or daily intake volumes of vitamin D. Therefore, serum calcium concentrations and daily intake volumes of vitamin D could not be used as adjustment variables. Third, sexual hormones, such as estrogens and testosterone, are an important determinant of serum 25(OH)D and ferritin. However, estrogens and testosterone were not employed in the KNHANES V-3 study (2012). The serum 25 (OH)D for each season, along with calcium, hepcidin, estrogens, and testosterone levels, should be included as variables for serum 25(OH)D in future studies. Although the present study has these limitations, this is the first reported study to determine gender differences in the relationship between ferritin and vitamin D in Korean adults. Therefore, more accurate results might be obtained by performing a cohort study by adding these variables.

## Conclusion

The present study investigated the association between serum ferritin levels and vitamin D using data from the fifth KNHANES conducted in 2012. Serum 25(OH)D level was inversely associated with serum ferritin levels in men. Conversely, serum 25(OH)D level was positively associated with serum ferritin levels in premenopausal women and was not associated with serum ferritin levels in postmenopausal women.

## Supporting information

S1 Table Comparisons of vitamin D status and iron related indices according to serum ferritin quartiles in men. (DOCX)

S2 Table Comparisons of vitamin D status and iron related indices according to serum ferritin quartiles in premenopausal women. (DOCX)

S3 Table Comparisons of vitamin D status and iron related indices according to serum ferritin quartiles in postmenopausal women. (DOCX)
