# Supplementary material for: Gender difference in relationship between serum ferritin and 25-hydroxyvitamin D in Korean adults
Source: PLoS One. 2017 May 31;12(5):e0177722. doi: 10.1371/journal.pone.0177722 (PMC5451000; doi:10.1371/journal.pone.0177722)
Supplement: S1 Table — (DOCX) [file pone.0177722.s001.docx]

**Supplement 1 Comparisons of vitamin D status and iron related indices according to serum ferritin quartiles in men**

| Variables | Category | Serum ferritin levels (μg/L) | | | | *P-*value |
| --- | --- | --- | --- | --- | --- | --- |
|  |  | Quartile 1 (n = 541)  (< 61.36 μg/L) | Quartile 2 (n = 540)  (≥ 61.36, < 98.99 μg/L) | Quartile 3 (n = 541)  (≥ 98.99, < 153.59 μg/L) | Quartile 4 (n = 540)  (≥ 153.59 μg/L) |  |
| Ferritin (μg/L) |  | 39.21 ± 15.90 | 80.38 ± 10.77 | 122.54 ± 54.55 | 222.91 ± 59.59 | < 0.001 |
| 25(OH)D (ng/mL) |  | 18.15 ± 5.72 | 18.18 ± 5.74 | 18.26 ± 5.53 | 17.16 ± 5.40 | 0.003 |
|  | **< 10.0 (n/%)** | 24/4.4% | 22/4.1% | 21/3.9% | 38/7.0% | 0.009 |
|  | **≥ 10.0, < 20.0 (n/%)** | 330/61.0% | 337/62.4% | 344/63.6% | 363/67.2% |  |
|  | **≥ 20.0 (n/%)** | 187/34.6% | 181/33.5% | 176/32.5% | 139 /25.7% |  |
| Metabolic syndrome **(n/%)** |  | 92/17.0% | 111/20.6% | 134/24.8% | 181/33.5% | < 0.001 |
| Age (years) |  | 54.17 ± 16.12 | 50.61 ± 16.73 | 49.19 ± 15.52 | 50.65 ± 15.46 | < 0.001 |
| Fe (μg/dL) |  | 120.76 ± 53.41 | 131.09 ± 46.96 | 133.04 ± 49.97 | 134.94 ± 49.78 | < 0.001 |
| TIBC (μg/dL) |  | 329.00 ± 45.28 | 305.98 ± 34.30 | 304.29 ± 36.01 | 302.32 ± 38.74 | < 0.001 |
| TFS (%) |  | 37.34 ± 16.37 | 43.03 ± 15.08 | 43.89 ± 16.20 | 44.87 ± 16.13 | < 0.001 |
| Hb (g/dL) |  | 14.86 ± 1.50 | 15.28 ± 1.05 | 15.43 ± 1.03 | 15.43 ± 1.28 | < 0.001 |
| Hct (%) |  | 44.07 ± 3.88 | 44.96 ± 2.88 | 45.30 ± 2.82 | 45.19 ± 3.33 | < 0.001 |
| MCV (fL) |  | 92.27 ± 5.06 | 93.25 ± 3.87 | 92.87 ± 3.69 | 93.42 ± 4.31 | < 0.001 |

25(OH)D: 25-hydroxyvitamin D, Fe: serum iron, TIBC: total iron binding capacity, TFS: transferrin saturation, Hb: hemoglobin, Hct: hematocrit, MCV: mean corpuscular volume.
