# Supplementary material for: Gender difference in relationship between serum ferritin and 25-hydroxyvitamin D in Korean adults
Source: PLoS One. 2017 May 31;12(5):e0177722. doi: 10.1371/journal.pone.0177722 (PMC5451000; doi:10.1371/journal.pone.0177722)
Supplement: S2 Table — (DOCX) [file pone.0177722.s002.docx]

**Supplement 2 Comparisons of vitamin D status and iron related indices according to serum ferritin quartiles in premenopausal women**

| Variables | Category | Serum ferritin levels (μg/L) | | | | *P-*value |
| --- | --- | --- | --- | --- | --- | --- |
|  |  | Quartile 1 (n = 391)  (< 10.34 μg/L) | Quartile 2 (n = 391)  (≥ 10.34, < 24.46 μg/L) | Quartile 3 (n = 410)  (≥ 24.46, < 43.75 μg/L) | Quartile 4 (n = 371)  (≥ 43.75 μg/L) |  |
| Ferritin (μg/L) |  | 5.31 ± 2.49 | 17.05 ± 4.12 | 34.00 ± 5.82 | 75.66 ± 33.85 | < 0.001 |
| 25(OH)D (ng/mL) |  | 14.26 ± 4.70 | 15.03 ± 4.79 | 14.77 ± 4.53 | 15.83 ± 5.11 | < 0.001 |
|  | **< 10.0 (n/%)** | 64/16.4% | 49/12.6% | 56/13.6% | 36/9.7% | 0.002 |
|  | **≥ 10.0, < 20.0 (n/%)** | 291/74.4% | 291/74.4% | 305/74.4% | 266/71.7% |  |
|  | **≥ 20.0 (n/%)** | 36/9.2% | 51/13.0% | 49/12.0% | 69/18.6% |  |
| Metabolic syndrome **(n/%)** |  | 41/10.5% | 35/9.0% | 54/13.2% | 79/21.3% | < 0.001 |
| Age (years) |  | 37.38 ± 9.18 | 38.32 ± 11.24 | 39.50 ± 13.55 | 43.54 ± 16.26 | < 0.001 |
| Fe (μg/dL) |  | 67.86 ± 42.77 | 111.12 ± 45.17 | 108.20 ± 42.27 | 114.76 ± 44.32 | < 0.001 |
| TIBC (μg/dL) |  | 382.56 ± 50.94 | 329.59 ± 42.11 | 310.41 ± 37.83 | 300.71 ± 33.99 | < 0.001 |
| TFS (%) |  | 18.31 ± 12.05 | 34.06 ± 13.95 | 35.17 ± 13.85 | 38.30 ± 14.72 | < 0.001 |
| Hb (g/dL) |  | 11.89 ± 1.46 | 13.12 ± 0.86 | 13.23 ± 0.90 | 13.41 ± 0.88 | < 0.001 |
| Hct (%) |  | 36.89 ± 3.51 | 39.46 ± 2.38 | 39.52 ± 0.57 | 40.02 ± 2.46 | < 0.001 |
| MCV (fL) |  | 86.27 ± 7.64 | 91.56 ± 3.99 | 92.05 ± 3.61 | 92.51 ± 3.52 | < 0.001 |

25(OH)D: 25-hydroxyvitamin D, Fe: serum iron, TIBC: total iron binding capacity, TFS: transferrin saturation, Hb: hemoglobin, Hct: hematocrit, MCV: mean corpuscular volume.
