# Supplementary material for: Gender difference in relationship between serum ferritin and 25-hydroxyvitamin D in Korean adults
Source: PLoS One. 2017 May 31;12(5):e0177722. doi: 10.1371/journal.pone.0177722 (PMC5451000; doi:10.1371/journal.pone.0177722)
Supplement: S3 Table — (DOCX) [file pone.0177722.s003.docx]

**Supplement 3 Comparisons of vitamin D status and iron related indices according to serum ferritin quartiles in postmenopausal women**

| Variables | Category | Serum ferritin levels (μg/L) | | | | *P-*value |
| --- | --- | --- | --- | --- | --- | --- |
|  |  | Quartile 1 (n = 356)  (< 35.55 μg/L) | Quartile 2 (n = 355)  (≥ 35.55, < 57.56 μg/L) | Quartile 3 (n = 356)  (≥ 57.56, < 85.80 μg/L) | Quartile 4 (n = 355)  (≥ 85.80 μg/L) |  |
| Ferritin (μg/L) |  | 23.04 ± 8.56 | 46.34 ± 6.32 | 70.37 ± 7.87 | 127.56 ± 7.88 | < 0.001 |
| 25(OH)D (ng/mL) |  | 17.56 ± 5.93 | 17.31 ± 5.69 | 17.45 ± 5.89 | 17.91 ± 6.16 | 0.571 |
|  | **< 10.0 (n/%)** | 30/8.4% | 25/7.0% | 23/6.5% | 20/5.6% | 0.879 |
|  | **≥ 10.0, < 20.0 (n/%)** | 220/61.8% | 226/63.7% | 228/64.0% | 229/64.5% |  |
|  | **≥ 20.0 (n/%)** | 106/29.8% | 104/29.3% | 105/29.5% | 106/29.9% |  |
| Metabolic syndrome **(n/%)** |  | 136/38.2% | 135/38.0% | 148/41.6% | 170/47.9% | 0.025 |
| Age (years) |  | 65.14 ± 10.02 | 64.76 ± 9.14 | 63.62 ± 8.57 | 63.93 ± 9.49 | 0.226 |
| Fe (μg/dL) |  | 93.73 ± 34.26 | 102.36 ± 30.44 | 102.85 ± 32.83 | 111.59 ± 35.69 | < 0.001 |
| TIBC (μg/dL) |  | 334.61 ± 44.92 | 316.12 ± 35.63 | 307.12 ± 37.76 | 298.41 ± 37.45 | < 0.001 |
| TFS (%) |  | 28.65 ± 11.40 | 32.90 ± 10.31 | 33.49 ± 10.53 | 37.70 ± 12.36 | < 0.001 |
| Hb (g/dL) |  | 12.94 ± 1.24 | 13.31 ± 0.90 | 13.38 ± 0.91 | 13.40 ± 1.03 | < 0.001 |
| Hct (%) |  | 39.20 ± 3.22 | 40.02 ± 2.66 | 40.06 ± 2.58 | 39.98 ± 2.94 | < 0.001 |
| MCV (fL) |  | 91.86 ± 4.87 | 93.25 ± 3.92 | 92.93 ± 3.73 | 93.03 ± 3.76 | < 0.001 |

25(OH)D: 25-hydroxyvitamin D, Fe: serum iron, TIBC: total iron binding capacity, TFS: transferrin saturation, Hb: hemoglobin, Hct: hematocrit, MCV: mean corpuscular volume.
